# Knowledge, attitude and skills before and after a module on pharmaceutical promotion in a Nepalese medical school

**DOI:** 10.1186/1756-0500-5-8

**Published:** 2012-01-06

**Authors:** P Ravi Shankar, Kundan K Singh, Rano M Piryani

**Affiliations:** 1 Department of Clinical Pharmacology, KIST Medical College, P.O. Box 14142, Kathmandu, Nepal; 2 Department of Clinical Pharmacology, KIST Medical College, Lalitpur, Nepal; 3 Department of Medicine, KIST Medical College, Lalitpur, Nepal

## Abstract

**Background:**

Pharmaceutical promotion is widespread and can impact prescribing by health professionals. Little research has been conducted on interactions between medical students and the pharmaceutical industry. Teaching about pharmaceutical promotion is inadequate. A survey showed that many schools spend only about two hours teaching this important topic while others spend around six hours. Recently a manual on understanding and responding to promotion has been published by Health Action International (HAI) and the World Health Organization (WHO). From April to August 2011 the department of Clinical Pharmacology at KIST Medical College, Lalitpur, Nepal conducted a module on pharmaceutical promotion for second year students based on the manual. The module used active learning strategies such as brainstorming sessions, role plays and group activities. The study worked on the hypothesis that a module on pharmaceutical promotion will be effective in improving the knowledge, attitude and skills of medical students regarding pharmaceutical promotion. The impact of the module on knowledge, attitude and skills was tested using a retrospective-pre questionnaire. The scores according to gender and method of financing of medical education before and after the module were compared using appropriate non-parametric tests.

**Results:**

Eighty-seven of the 100 second year students (87%) participated in the study. 47 were females (54%) and 39 (44.83%) were males and one did not state the gender. Seventy-seven students (88.5%) were self-financing while 9 were scholarship students. The median knowledge, attitude and skills score before the module were 9, 13 and 6 respectively while the overall score was 28. The scores increased significantly to 16, 15 and 14 respectively after the module while the overall score increased to 45. The median attitude scores and total scores were significantly higher among females both before and after the module. The scores did not vary with method of financing of medical education. All scores increased significantly at the end of the module.

**Conclusions:**

The nine hour module held over a period of four months was effective in improving respondents' knowledge, attitudes and skills about pharmaceutical promotion. The module was not resource intensive and used resources already available in the institution. Similar modules can be considered in other medical and health profession schools in Nepal, South Asia and other developing countries.

## Background

Pharmaceutical promotion has been described as 'all informational and persuasive activities by manufacturers and distributors, the effect of which is to induce the prescription, supply, purchase and/or use of medicinal drugs [1].' In recent years links between doctors and the pharmaceutical industry have grown enormously. A large survey conducted in the United States (US) found that over 90% of physicians reported some type of relationship with the pharmaceutical industry [2]. Companies spent US$ 57.5 billion on promotion in 2004 in the US [3].

A recent article states that little research has been conducted on the relationship between the pharmaceutical industry and medical students [4]. The authors state students receiving gifts can be subject to reciprocal obligations which can bias their future prescribing. Medical educators, the authors believe, have a duty to protect students from industry influence. A survey conducted in 2005 showed that although many medical and pharmacy schools around the world included pharmaceutical promotion in their curriculum, many spent less than a day and most devoted only one or two hours on this important topic [5]. The manual developed by the World Health Organization (WHO) and Health Action International (HAI) recently on 'Understanding and responding to pharmaceutical promotion' [6] serves as a companion module to the WHO book 'Guide to good prescribing' and will assist teachers to teach students about promotion.

The department of Clinical Pharmacology at KIST Medical College (KISTMC) has the objective of teaching students to use essential medicines rationally. During practical sessions conducted in small groups students learn about essential medicines, analyzing prescriptions, selecting P-drugs, communicating with simulated patients, solving clinical problems, learning about reporting adverse drug reactions and critically analyzing pharmaceutical promotion [7]. Promotional activities are increasing in Nepal and medical conferences are supported by the industry [8]. Industry also sponsors student events. Among the events sponsored are welcome programs for fresh batches of students, farewell parties of the final batch and informal student get togethers. Sponsorship is usually in the form of financial support for the events. We conducted a module on promotion based on the WHO and HAI manual, building on the topics covered during the pharmacology practical classes. We decided to test the impact of the module on knowledge, attitude and skills of participants using the retrospective pre then post design questionnaire. This is a popular method to assess learners' self-reported changes in different domains, takes little time, is minimally intrusive and avoids pre-test sensitivity and response shift bias [9,10]. In this method information is collected only once, at the end of the program. The participants are asked to rate their knowledge, attitude, skill, behavior at the end of the program and then reflect back and rate those same qualities before participating in the program.

The hypothesis of the study was: A module on pharmaceutical promotion will be effective in improving the knowledge, attitude and skills of medical students regarding pharmaceutical promotion.

The present study was carried out with the following objectives:

a To obtain participants' perceptions about their knowledge, attitude and skills related to pharmaceutical promotion before and after the promotion module

b To compare the scores according to gender and method of financing of medical education and

c To compare scores before and after the module.

## Methods

The module titled 'The Skeptic Doctor' was held on Mondays during early clinical exposure from April to early August 2011. The 100 students of the 2009 batch were divided into two batches of 50 students each; two parallel sessions were held on alternate Mondays from 8 05-9 10 am. The batches were further divided into small groups of 10 students each; each batch being further subdivided into five small groups of 10 students. Facilitator presentations, case scenarios, brain storming sessions, group activities and role plays were the teaching/learning methods/strategies used. The module was developed based on the book 'Understanding and responding to pharmaceutical promotion.' Two of the authors, PRS and RMP acted as facilitators for the small group sessions. Students facilitated some of the sessions under the guidance and supervision of the faculty. The topics addressed during the facilitator presentations included 'Tension between health and commercial aims', ' Analyzing advertisements in medical journals concentrating on text, pictures, tables and graphs', 'Medical representatives', 'Medical students and the pharmaceutical industry', 'Independent sources of medicine information' and 'Evaluating the quality of health information on the internet'. The case scenarios and group activities dealt with criteria for assessing the quality of health information on the internet, reasons why doctors should carefully consider whether or not to accept free drug samples, students being offered sponsorship and gifts by the pharmaceutical industry, analyzing pharmaceutical advertisements in medical journals, analyzing advertisements directed at consumers in television, analyzing company sponsored internet blogs. The role plays dealt with a medical representative (MR) promoting a medicine to a doctor and the onlookers critically analyzing the presentation using a checklist and issues linked with industry-doctor relationship. In a parallel session, the other batch engaged with assignments and group exercises based on the manual under the supervision and guidance of the author KKS.

The sessions conducted were 'Promotion of medicines and public health', 'Techniques that influence the use of medicines', 'Analyzing pharmaceutical advertisements in medical journals', 'Pharmaceutical sales representatives', 'Promotion to consumers', 'Students and the pharmaceutical industry', and 'Using unbiased prescribing information'. Students critically analyzed offers by the pharmaceutical industry to promote different student events and supply free textbooks and other study equipment. They also developed criteria to assess the quality of health websites on the internet. Facilitator inputs were followed by discussion in each session. The criteria developed by the French drug bulletin 'La revue Prescrire' to analyze information provided by MRs was used to critically analyze role plays. Ethical issues concerning the relationship between pharmaceutical companies and doctors were explored using role plays.

Participant perception about the influence of the module on knowledge, attitude and skills was measured using a retrospective pre questionnaire developed by the authors. The study was approved by the Institutional Review Board of KIST Medical College. The statements to be included in the questionnaire were developed based on points covered during the module and highlighted in the manual after discussion among the authors. The questionnaire was pilot tested for comprehensibility among three third year students. The retrospective pre questionnaire used is shown as an additional file 1. Gender and method of financing of medical education were noted. Differences in scores according to gender have been noted by the authors in previous studies. The college admits two categories of students - (i) scholarship students selected on the basis of ranks obtained in an entrance examination conducted by Ministry of Education and (ii) self-financing students who have to pass (obtaining 50% marks) in an examination conducted by the University. Scholarship students are on the whole stronger academically than self-financing ones. Scores were allotted to various categories. The three categories for 'Knowledge' were 'No idea', 'Have a vague idea' and 'Clear idea'. The score for 'No idea' was 1, 2 for 'Have a vague idea', and 3 for 'Clear idea'. Six areas were explored under the heading 'Knowledge', giving a total score of 18.

For 'Attitudes' respondents' agreement with a set of five statements was explored using a Likert-type scale. The scores were: 5 = strongly agree, 4 = agree, 3 = no opinion, 2 = disagree and 1 = strongly disagree with the statement. The scores were reversed for negative statements. The scores for five statements were added to obtain the total 'Attitude' score. The maximum possible score was 25.

Five sets of 'Skills' were studied. For each skill the respondent was given the following choices: 'he/she was not confident', 'somewhat confident', 'very confident' in performing the skill and 'will be able to do independently in future'. These were given the scores 1, 2, 3 and 4 respectively. The total skills score was calculated adding the scores of the five skills. The maximum score was 20.

The total scores before and after the module was obtained by adding the 'Knowledge', 'Attitude' and 'Skills' scores. The maximum total score was 63. The median total 'Knowledge', 'Attitude', 'Skills', overall scores, and interquartile range were calculated. The data was analyzed using SPSS version 17 for Windows. One sample Kolmogorov-Smirnov test was used to determine the normality of the variables. Most variables were found not to follow a normal distribution and hence non-parametric tests were used. Median scores were compared according to gender and method of financing of medical education before and after the module, using the Mann-Whitney test. Median scores before and after the module were compared using the Wilcoxon signed ranks test. A p value less than 0.05 was taken as statistically significant.

## Results

Eighty-seven of the 100 second year students (87%) participated in the study. Forty-seven were female (54%) and 39 (44.83%) were male. Seventy-seven (88.5%) were self-financing while nine were scholarship students. One respondent each did not mention their gender and method of financing of medical education.

The median (interquartile range) Knowledge, Attitude and Skills scores before the module were 9 (3), 13 (3) and 6 (2) respectively. The maximum scores were 18, 25 and 20 respectively. The median total score before the module was 28 (6) [maximum score 63]. The median (interquartile range) Knowledge, Attitude and Skills scores after the module were 16 (2), 15 (4) and 14 (5) respectively. The median total score after the module was 45 (8). The knowledge scores for the topics 'Techniques used to promote medicines', 'Using unbiased information about drugs' and 'Physician-industry relationship' was 1 before the module. Median 'Knowledge' scores of all topics after the module were 3. The 'Attitude' scores for statements about 'accepting a pen from a drug company', 'the primary objective of the industry being to sell drugs' and about 'the benefits of seeing MRs' were low before the module. Scores increased after the module. The median 'Skills' scores for analyzing drug information, analyzing MR presentations, using statistics in critical appraisal, using independent information sources and educating consumers about promotion were 1 at the beginning of the module. The scores increased to 3 for analyzing drug information, MR presentations, and educating consumers. Scores were 2 for the other skills. Table 1 shows the 'Knowledge', 'Attitude' and 'Skills' scores as noted by the participants at the beginning of the module. The median attitude and total scores were significantly higher among female students. There were no differences in scores according to method of financing of medical education. Table 2 shows the 'Knowledge', 'Attitude' and 'Skills' scores as noted by the participants on completion of the module. The median and total 'Attitude' scores continued to be significantly higher among female students. There were no differences in scores according to method of financing of medical education.

**Table 1:** Knowledge, attitude and skills of participants at the beginning of the module on pharmaceutical promotion according to gender and method of financing of medical education

Characteristics		Median knowledge score (Interquartile range) (Maximum 18)	Median attitude score (Interquartile range) (Maximum 25)	Median skills score (Interquartile range) (Maximum 20)	Median total score (Interquartlie range) (Maximum 63)
Gender	Male	9 (3)	**11 (4)**	6 (2)	**27 (5)**
	
	Female	9 (2)	**13 (2)**	6 (2)	**28 (5)**
	
	P value	0.784	**< 0.001**	0.612	**0.038**

Method of financing	Self	9 (3)	13 (3)	8 (2)	28 (5.5)
	
	Scholar-ship	9 (2.5)	11 (4)	7 (3.5)	29 (7.5)
	
	P value	0.920	0.381	0.303	0.566

**Table 2:** Knowledge, attitude and skills of participants at the completion of the module on pharmaceutical promotion according to gender and method of financing of medical education

Characteristics		Median knowledge score (Interquartile range) (Maximum 18)	Median attitude score (Interquartile range) (Maximum 25)	Median skills score (Interquartile range) (Maximum 20)	Median total score (Interquartlie range) (Maximum 63)
Gender	Male	15 (3)	**15 (5)**	14 (4)	**42 (8)**

	Female	16 (2)	**16 (3)**	13 (5)	**46 (5)**

	P value	0.240	**0.001**	0.918	**0.013**

Method of financing	Self	16 (2)	16 (3.5)	14 (4)	45 (7.5)

	Scholar-ship	17 (2.5)	15 (7.5)	12 (7)	46 (8.5)

	P value	0.400	0.675	0.522	0.893

Table 3 compares the median 'Knowledge', 'Attitude', 'Skills' and total scores at the beginning and end of the module. The scores were significantly higher at the end of the module.

**Table 3:** Median scores at the beginning and end of the module on pharmaceutical promotion

Characteristic	Median score (Interquartile range)		P value
	
	Pre	Post	
Knowledge	**9 (3)**	**16 (2)**	**< 0.001**

Attitude	**13 (3)**	**15 (4)**	**< 0.001**

Skills	**6 (2)**	**14 (5)**	**< 0.001**

Total	**28 (6)**	**45 (8)**	**< 0.001**

## Discussion

All categories of scores and the total score increased significantly at the end of the module. Certain categories of scores varied according to gender before and after the module.

Sessions on promotion have been conducted in medical schools. In the US an innovative workshop was held for all third year medical students. Two faculty members and a pharmaceutical company representative (PCR) led the workshop which highlighted physician-PCR interactions [11]. Using a Likert scale, surveys conducted before and after the workshop elicited information from participants about different aspects of the interaction. Changes in attitudes were noted after the workshop. At Jefferson University, US, a series of four seminars was conducted with third year students where students were given articles dealing with physician-industry interactions, asked to summarize the same and present it to other students [12]. A faculty member moderated the discussion. Students were more cautious about industry interactions after the seminar series.

The median attitude score towards pharmaceutical promotion was higher among female students both before and after the module. According to our experience female students are more disciplined and show greater application to their studies. Another possible reason is that female students are less likely to go against the attitudes and opinions of the teachers and are more likely to emulate the same. Women students had a more cautious attitude towards the industry. The increase in Attitude scores among male students at the end of the module was however higher.

The knowledge of the topics 'Techniques used to promote medicines', 'Using unbiased information about drugs' and 'Physician-industry relationship' were low at the beginning of the module. The WHO/HAI manual describes in detail the techniques used in promotion and also mentions different unbiased sources of information about drugs. The high initial scores among our students about the importance of promotion and promotion of medicines to consumers may be partly attributed to the inputs they receive during pharmacology practical sessions. All median scores increased to 3 (clear idea) after the module.

The agreement with the statement about 'not accepting a pen from the industry' was low before the module. A pen with the name of a drug company's medicine may serve as a constant reminder to prescribe the medicine [6]. An organization called 'No free lunch' runs a pen amnesty programme where doctors can hand in their drug company pens and receive a 'No free lunch' pen in return [13]. The American Medical Student association (AMSA) as part of its PharmFree campaign runs a nationwide pen amnesty program.

Seeing and interacting with a MR is very common. Companies spend more than 25% of their marketing budgets on MRs [14]. MRs may provide information useful to physicians, may provide items like medicine samples which can be given to patients who cannot afford them and provide gifts. An author state MRs raise awareness of untreated conditions and may be doing good to society [15]. Another authority states that doctors should not see MRs both for reasons of professional integrity and sensible time management [16].

We were a bit surprised at students rating their skill of analyzing drug advertisements at 1 before the start of the module. The manual however describes analysis of a drug advertisement from many other aspects like pictures, graphs and claims in the advertisement and students may have felt that compared to the end of the module their knowledge at the beginning was low. Critical appraisal of clinical trials is an important skill for a doctor and requires some knowledge of statistics. In Nepal, Statistics is taught by the Department of Community Medicine mainly from a public health perspective. A session on critical appraisal was conducted at Manipal College of Medical Sciences, Pokhara, Nepal [17] during which problems were noted due to students' poor knowledge of statistics. Teaching of critical appraisal of evidence has been carried out in many centers in developed nations. In the US students learned about appraisal of randomized clinical trials by conducting an in-class randomized trial [18]. In Ireland a prospective controlled study was done of the impact of a 6 hour workshop on the ability of medical students to critically appraise literature [19]. The authors concluded that the workshop was effective in improving skills. Changes in the teaching of Statistics may be required. Statistics from the viewpoint of clinical research may need to be taught. Students use independent information sources during pharmacology practical sessions. They may not however consider sources such as text books as independent medicine information sources. We are thinking about making the teaching of this important skill more effective. In Nepal many institutions have free access to biomedical journals through the HINARI service of WHO. We are spreading awareness about HINARI and how to use it, during our session on independent sources of medicine information.

A recent study conducted at four US Medical schools concluded that education about pharmaceutical marketing practices and more restrictive policies governing industry-student relationship increased medical students' skepticism about such relationships and marketing practices and disapproval of industry representatives in the learning environment [20]. A cross-sectional survey conducted in the US concluded that health professions students' knowledge and attitudes towards the pharmaceutical industry are formed before graduation and professional curricula should address the influence of sales representatives before postgraduate training [21].

The strength of our study was the high response rate.

The study also had limitations. The questionnaire used was developed by the authors and the points to be included were developed through consensus. This was the first time we were using a retrospective-pre questionnaire for a study. We explained to students about the philosophy behind this questionnaire and how to answer queries. However, certain students may have faced difficulties though the authors were present to provide support when asked.

The scoring system was developed by the authors in consultation with a biostatistician. The nature of the questionnaire may have influenced student responses. The Knowledge, Attitude and Skills both before and after the module were studied at the end of the module. The facilitators strongly projected an attitude of independence from the pharmaceutical industry. The facilitators' attitudes and the emphasis and focus of the session may have influenced student responses. At the same time facilitators encouraged students to express their opinions freely. The anonymity of respondents was maintained. We have conducted questionnaire based studies previous to this, and have been successful in obtaining free and frank responses from the students.

## Conclusions

The nine hour module held over a period of four months was effective in improving respondents' Knowledge, Attitudes and Skills about pharmaceutical promotion. The module based on the HAI/WHO manual used small group learning strategies. The module was not resource intensive, using resources already available in the institution. Similar modules can be considered in other medical and health profession schools in Nepal, South Asia and other developing countries.

## Conflicts of interests

The authors declare that they have no competing interests.

## Authors' contributions

PRS was involved in conducting the module, conceptualizing the study, designing the questionnaire, collecting the data, reviewing the literature and writing the manuscript. KKS helped in conducting the module, conceptualizing the study, designing the questionnaire, collecting the data, reviewing the literature and writing the manuscript. RMP helped in conducting the module, conceptualizing the study and writing the manuscript. All authors have read and approved the final submitted version of the manuscript.

## Supplementary Material

Additional file 1 **Influence of pharmaceutical promotion - A Retrospective Pre (DOC 57 kb)**. Click here for file
