# Supplementary material for: Knowledge, attitude and skills before and after a module on pharmaceutical promotion in a Nepalese medical school
Source: BMC Res Notes. 2012 Jan 6;5:8. doi: 10.1186/1756-0500-5-8 (PMC3284404; doi:10.1186/1756-0500-5-8)
Supplement: Additional file 1 — Influence of pharmaceutical promotion - A Retrospective Pre (DOC 57 kb). [file 1756-0500-5-8-S1.DOC]

Influence of pharmaceutical promotion – A Retrospective Pre

Gender: Method of financing of education:

| **Knowledge** | **Before the module** | | | | | | | | **After the module** | | | | | | | | |
| --- | --- | --- | --- | --- | --- | --- | --- | --- | --- | --- | --- | --- | --- | --- | --- | --- | --- |
|  | **No idea** | | **Have a vague idea** | | | **Clear idea** | | | **No idea** | **Have a vague idea** | | | | | **Clear idea** | | |
| Promotion of medicines & public health |  | |  | | |  | | |  |  | | | | |  | | |
| Techniques used to promote medicines |  | |  | | |  | | |  |  | | | | |  | | |
| Promotion of medicines to consumers |  | |  | | |  | | |  |  | | | | |  | | |
| Medical student & the pharma industry |  | |  | | |  | | |  |  | | | | |  | | |
| Using unbiased information about drugs |  | |  | | |  | | |  |  | | | | |  | | |
| Physician-industry relationship |  | |  | | |  | | |  |  | | | | |  | | |
| **Attitudes** | **Strongly**  **Agree** | | **Agree** | **No**  **opinion** | | **Disagree** | | **Strongly**  **disagree** | **Strongly**  **Agree** | | **Agree** | **No**  **opinion** | | **Disagree** | | | **Strongly**  **disagree** |
| The industry’s primary objective is to sell their medicines. |  | |  |  | |  | |  |  | |  |  | |  | | |  |
| I will accept expensive gifts from the industry |  | |  |  | |  | |  |  | |  |  | |  | | |  |
| Accepting a pen from a drug company is OK. |  | |  |  | |  | |  |  | |  |  | |  | | |  |
| Pharma industry should not sponsor medical student events. |  | |  |  | |  | |  |  | |  |  | |  | | |  |
| Seeing a MR is of great benefit to doctors. |  | |  |  | |  | |  |  | |  |  | |  | | |  |
| **Skills** | **Not**  **confident** | **Somewhat**  **confident** | | | **Very**  **confident** | | **Will be able to do independently in future** | | **Not**  **confident** | | **Somewhat**  **confident** | | **Very**  **confident** | | | **Will be able to do**  **independently**  **in future** | |
| Analyzing drug advertisements |  |  | | |  | |  | |  | |  | |  | | |  | |
| Analyzing MR presentations |  |  | | |  | |  | |  | |  | |  | | |  | |
| Using statistics in critical appraisal |  |  | | |  | |  | |  | |  | |  | | |  | |
| Using independent information sources information |  |  | | |  | |  | |  | |  | |  | | |  | |
| Educating consumers about promotion |  |  | | |  | |  | |  | |  | |  | | |  | |

**The Skeptic Doctor 2011**

**Department of Medical Education, KISTMC**
